# Examining the relationship between the Short Warwick-Edinburgh Mental Well-being Scale (SWEMWBS) and EQ-5D-5L and comparing their psychometric properties

**DOI:** 10.1186/s12955-023-02108-y

**Published:** 2023-03-16

**Authors:** Yanming Hong, Xinru Jiang, Tiantian Zhang, Nan Luo, Zhihao Yang

**Affiliations:** 1 The Third Affiliated Hospital of Sun Yat-Sen University, Guangzhou, People’s Republic of China; 2 Tianhe Foreign Language School, Guangzhou, People’s Republic of China; 3 College of Pharmacy, Jinan University, Guangzhou, People’s Republic of China; 4 Saw Swee Hock School of Public Health, National University of Singapore, Singapore, Singapore; 5 Health Services Management Department, Guizhou Medical University, Guiyang, People’s Republic of China

**Keywords:** EQ-5D-5L, Health-related quality of life, SWEMWBS, Mental well-being, China

## Abstract

**Background:**

The purpose of this study is to examine the relationship between the Short Warwick-Edinburgh Mental Well-being Scale (SWEMWBS) and EQ-5D-5L and compare their psychometric properties in 4 chronic conditions in China.

**Methods:**

Participants were invited to complete the online survey. Spearman’s rank correlation was used to evaluate the correlation between SWEMWBS and EQ-5D-5L; exploratory factor analysis was used to ascertain the number of unique underlying latent factors measured by SWEMWBS and EQ-5D-5L. Next, we assessed the psychometric properties of SWEMWBS and EQ-5D-5L by reporting distributions and examining their known-group validity and convergent validity.

**Results:**

In total, 500 individuals participated the online survey. Spearman’s rank correlation showed that EQ-5D-5L dimensions, except for the anxiety/depression dimension, were weakly correlated with all dimensions of SWEMWBS. The two-factor solution for exploratory factor analysis found that all of SWEMWBS dimensions loaded onto one factor, four EQ-5D-5L dimensions (mobility, self-care, usual activities and pain/discomfort) onto another, and the EQ-5D-5L item of anxiety/depression item loaded moderately onto both factors. Patients of four disease groups had different distributions of responses for both SWEMWBS and EQ-5D-5L. In terms of known-group validity, both the F statistic and AUROC value of EQ-5D-5L utility scores were significantly higher than SWEMWBS scores in all four pair-wised comparisons. The Pearson correlation coefficient between EQ-5D-5L utility scores, SWEMWBS scores and EQ-VAS was 0.44 (*P* < 0.01) and 0.65 (*P* < 0.01), respectively.

**Conclusions:**

SWEMWBS and EQ-5D-5L measure different constructs and can be seen as complementary measures. Both measures demonstrated good convergent validity and known-group validity with EQ-5D-5L being a more sensitive measure, even for mental conditions.

## Background

The EQ-5D-5L is a widely used health-related quality of life (HRQoL) instrument and its descriptive system comprises five dimensions: mobility (MO), self-care (SC), usual activities (UA), pain/discomfort (PD), and anxiety/depression (AD) [1]. The brevity of the EQ-5D-5L makes it a popular patient-reported outcome measure (PROM), in addition to its primary use as a tool to calculate health utility value [2]. Compared to other PROMs that can provide rich information on different aspects of HRQoL, EQ-5D-5L may not be comprehensive and may be insensitive in measuring mental health, especially the construct of positive mental health [3]. This is mainly because the five dimensions of EQ-5D-5L are more focused on physical and functioning health, with only one dimension of anxiety/depression directly measuring mental health problems. Positive mental health is defined as an optimal way of psychological functioning and a general feeling of well-being [4]. In contrast, negative mental health includes deleterious facets such as health problems, psychopathology or psychiatric disorders.

Mental well-being covers two perspectives: hedonic well-being relates to a subjective appraisal of life satisfaction, affective emotions and moods, while eudaimonic well-being focuses on individuals’ psychological functioning and self-actualization [5, 6]. The Warwick-Edinburgh Well-Being Scale (WEMWBS) was developed in 2007 and has been broadly used to measure positive mental well-being [7]. WEMWBS is a 14-item instrument, which assesses affective-emotional aspects, cognitive evaluative dimensions, and psychological functioning [8]. In 2009, a brief 7-item version, the Short Warwick–Edinburgh Mental Well-Being Scale (SWEMWBS) was developed using the Rasch modeling method [9]. The short version focuses more on functioning than subjective aspects of mental well-being, with fewer items covering hedonic well-being or affect. However, the SWEMWBS was found to have preferable psychometric properties than the full version given its robust measurement properties and with the advantage of its additional brevity, and has been widely used in many population studies globally [8]. SWEMWBS has been translated into Chinese, Swedish and Norwegian languages [10, 11] and its validity and reliability have been demonstrated in the general population and hospitalized patients with mental illness in Hong Kong [10, 12, 13] and other populations (e.g. deaf British sign language users, Norwegian adults, and adolescents, Swedish adults, people with schizophrenia, depression and anxiety spectrum disorders in Singapore [11, 14–16].

EQ-5D-5L measures both physical health and mental health, but there is only one item (i.e., anxiety/depression) measures the construct of mental health, it is not clear whether this item could measure positive mental health, as measured by SWEMWBS. For this reason, we conducted this head-to-head study to understand the relationship between EQ-5D-5L (a HRQoL measure) with SWEMWBS (a positive mental health measure). Moreover, compared to the large number of studies investigating population HRQoL using EQ-5D-5L [17, 18], the number of studies evaluating the population’s mental well-being using SWEMWBS is scarce in China [11–13]. Studies have shown that positive mental well-being can affect health and social outcomes [19, 20]. In this study, we aimed to examine the relationship between the SWEMWBS and EQ-5D-5L and compared their psychometric properties in individuals with 4 chronic conditions including chronic hepatitis B (CHB), depression, generalized anxiety disorder (GAD), and HIV/AIDS in China.

## Methods

### Study design

This study utilized the psychometric survey data of the E-QALY project collected in China [21, 22]. The E-QALY project aims to develop a new generic measure that covers a broader quality of life construct, which is relevant to health, social care, and public health sectors [23]. The online survey includes a set of demographic questions, health condition status and caring experience, followed by 64 candidate E-QALY items, EQ-5D-3L, EQ-5D-5L and SWEMWBS. The sample size was fixed at 500 [24–26] considering the primary purpose of the data was used to conduct factor analysis and spearman correlation coefficients analysis for developing EQ-HWB [22]. This is a sufficient sample size for this study given that published EQ-5D-5L and SWEMWBS validation and comparison studies used a sample size of 500 or less [16, 27–30]. This data was collected between April and July 2019 online by Accent, a U.K. online survey company. Quotas and inclusion criteria were applied to recruit a sample of 500 participants who lived in China and were aged above 18, in which there were similar numbers of individuals with GAD, HIV/AIDS, CHB, or depression, or without any of those 4 chronic conditions. The study was approved by the Ethics Committee of University of Sheffield, United Kingdom (Approval letter number 025524) and the IRB of Jinan University, China (Approval letter number JNUKY-2020–001). Informed consent was obtained from all participants prior to the online survey.

The online survey began by giving an outline of the research purpose. Participants were then asked to report their disease history. Eligible respondents reported their background information including education level, gender, age, etc. Next, respondents were asked to respond to the core survey that includes the E-QALY candidate items, two versions of EQ-5D descriptive systems, EQ-VAS (only completed once) and SWEMWBS. This study utilized the background information, EQ-5D-5L, EQ-VAS and SWEMWBS data collected in the psychometric survey in China. The order of completing the SWEMWBS and EQ-5D-5L was also randomized with half of sample completing SWEMWBS first and the other half completing EQ-5D-5L first.

### Instruments

The EQ-5D-5L is a generic preference-based HRQoL instrument developed by the EuroQol Group. It was translated into simplified Chinese following a strict translation process [4] and its validity and reliability have been demonstrated in different health conditions [5–9] in China. It consists of a five-item descriptive system and a visual analog scale (EQ-VAS) [31, 32]. The descriptive system has five health dimensions, i.e., mobility, self-care, usual activities, pain/discomfort, anxiety/depression, and five response levels (1 = no problems, 2 = slight problems, 3 = moderate problems, 4 = severe problems and 5 = unable/extreme problems) for each dimension. An important characteristic of EQ-5D-5L is it allows the calculation of health utility values that reflect the desirability of a health state. In this study, EQ-5D-5L health utility values were calculated using the value set of China [33]. The EQ-VAS records the respondent’s current self-rated health on a 20-cm-long vertical thermometer-like scale from 0 (‘Worst imaginable health state’) to 100 (‘Best imaginable health state’).

The SWEMWBS is the short version of the Warwick-Edinburgh Mental well-being Scale (WEMWBS), which was developed to measure the mental well-being of the general population. The SWEMWBS consists of seven questions: I’ve been feeling optimistic about the future (OP), I’ve been feeling useful (USE), I’ve been feeling relaxed (RE), I’ve been dealing with problems well (PR), I’ve been thinking clearly (CL), I’ve been feeling close to other people (CLO), and I’ve been able to make up my mind about things (MI) and each question includes five frequency options (1 = none of the time, 2 = rarely, 3 = some of the time, 4 = often and 5 = all of the time) [9]. The Simplified Chinese translation was obtained from the developer of WEMWBS, which was translated by Dong et al. [34]. Raw level summary score (LSS) was summed and converted to metric total score using the SWEMWBS conversion table [9].

Note the response levels reversed between SWEMWBS and EQ-5D-5L on item level, with a higher response indicating better results for SWEMWBS but worse results for EQ-5D-5L. On aggregate level, higher score suggests better results for both EQ-5D-5L utility value, EQ-VAS and SWEMWBS overall score. In addition, the recall periods differed as EQ-5D-5L uses ‘today’ and SWEMWBS uses ‘over the past two weeks’.

### Statistical analyses

We first described the characteristics of our sample and examined the relationship of the EQ-5D-5L and SWEMWBS. Spearman’s rank correlation was used to evaluate the association between the EQ-5D-5L dimensions and SWEMWBS dimensions. Exploratory factor analysis (EFA) was used to ascertain the number of unique underlying latent factors associated with the attributes assessed by the EQ-5D-5L and SWEMWBS. Secondly, we assessed their psychometric properties. The distributions of the EQ-5D-5L and SWEMWBS were reported. Specifically, items with over 70% of respondents reporting the best state and the worst state suggesting ceiling effect and floor effect respectively [35]. Known-group validity between healthy and each condition group was assessed for EQ-5D-5L utility, EQ-VAS and SWEMWBS score. Convergent validity was examined for EQ-5D-5L utility and SWEMWBS score using EQ-VAS as a benchmark. Data were analyzed using IBM SPSS Statistics for Windows, Version 22.0. Armonk, NY: IBM Corp (2013) and Mplus 8.3 Combo Version for Windows.

#### Association

Spearman’s rank correlation was used to evaluate the relationship between the EQ-5D-5L dimensions and SWEMWBS dimensions. Correlations were deemed as weak when scores fell between 0.10 and 0.29, moderate when between 0.30 and 0.49, and strong when greater than 0.5 [36–38]. Statistical significance was set at the 5% level. Since SWEMWBS measures mental well-being, we hypothesized that its dimensions have low correlations with EQ-5D-5L dimensions, except for anxiety/depression, which measures mental health.

#### Exploratory factor analysis

The purpose of exploratory factor analysis (EFA) is to reduce data dimensionality and to ascertain relatively few factors to describe the observed correlations among variables [39, 40]. In the EFA, data were sifted using the Kaiser–Meyer–Olkin (KMO) measure of sampling adequacy (> 0.5) and Bartlett’s test of sphericity (< 0.05) [41]. The KMO value ranged from 0 to 1, with greater than 0.60 considered suitable for factor analysis. The number of factors retained was selected according to the Kaiser Criterion [26], which claims retaining factors with eigenvalues bigger than 1 and using the scree plot to evaluate the suitability of this choice. The parallel analysis was run to ascertain the number of factors to be retained in model [42, 43]. We applied 1,000 random data sets to conduct the parallel analysis and then overlaid the results onto a single plot with the scree plot. Factors with eigenvalues in the observed data that are greater than the simulated data suggest “true” factors. Using an oblique Promax rotation allows for the potential that factors are correlated. Examining the rotated factor matrix, we identified items with pattern coefficients of 0.40 or greater as contributing to a factor and retained them. EFA was applied to all items from both the EQ-5D-5L (5 items) and the SWEMWBS (7 items).

#### Known-group validity

Known-group validity was evaluated by examining the mean, standard error (SE), median, and interquartile range (IQR) of EQ-5D-5L utility score, EQ-VAS and SWEMWBS score between healthy and each condition group. We hypothesized that the healthy group would have higher scores than the four disease groups. To investigate how the EQ-5D-5L and SWEMWBS perform in terms of discriminating between healthy and each condition group, the Mann–Whitney test was used to compare the distributions of the responses to the EQ-5D-5L and SWEMWBS dimensions. We listed the median values of each dimension as a reference. The efficiency of the EQ-5D-5L/SWEMWBS scores in differentiating between the known groups described above was tested using the F statistics based on the one-way analysis of variance [44–46]. F statistic has been used in previous studies as a way of comparing relative efficiency between two instruments [45–47]. The F statistic is defined as the ratio of intergroup variance dividing by intragroup variance, which is used for model-level significance tests in the linear regression model. When the model is significant, the value of the F statistic could be interpreted as the advantage of intergroup variance over intragroup variance. As the regression model is increasingly capable of capturing the change of regression target, the intergroup variance is increasingly dominant, and we will also expect a larger value of F statistic [48]. As a result, the index score with a higher F statistic would be supposed to be more efficient than its comparator because a greater value is much more likely to lead to statistical significance. As a complementary analysis, the efficiency of the EQ-5D-5L/SWEMWBS scores was also evaluated using the area under the receiver-operating characteristics curve (AUROC) [49]. The AUROC value ranges from 0.5 to 1.0, with a greater value suggesting better predictive ability.

#### Convergent validity

Convergent validity was examined for EQ-5D-5L utility and SWEMWBS score using EQ-VAS as a benchmark using the Pearson correlation coefficient, where the absolute value of Pearson correlation coefficient < 0.40 were considered as weak, moderate if between 0.40 and 0.70 and strong if > 0.70. Since the EQ-VAS fully evaluates the respondent’s overall state of health including physical health and mental health, we hypothesized that EQ-VAS has a positive correlation with the EQ-5D-5L utility scores and SWEMWBS scores.

## Results

In total, 500 individuals participated the online survey, including 140 healthy individuals, 122 individuals with CHB, 107 with depression, 90 individuals with GAD and 101 with HIV/AIDS. Some respondents reported multiple conditions, e.g. 68 individuals reported both depression and GAD. In general, the whole study sample was young. The gender proportions of the five groups were generally balanced except for the group of HIV/AIDS, in which, about 87.1% of individuals were female. In terms of the age distribution, the healthy group was mostly young (mean 31.02 years old, SD: 8.55); the CHB group had more participants aged between 40 and 49; the depression and GAD groups had individuals from all four age groups, and the HIV/AIDS group aged mainly from 30 to 49. Individuals with tertiary education accounted for over 80% for all four disease groups and the healthy group had more individuals with secondary education. Table 1 shows the demographic information by condition.

**Table 1:** Sample characteristics

Characteristics	Conditions					
	Total N (%)	Healthy (%)	CHB N (%)	Depression N (%)	GAD N (%)	HIV/AIDS N (%)
Subgroup sample size	500	140	122	107	90	101
Gender						
Male	200 (40)	68 (48.6)	53 (43.4)	56 (52.3)	42 (46.7)	13 (12.9)
Female	300 (60)	72 (51.4)	69 (56.6)	51 (47.7)	48 (53.3)	88 (87.1)
Age group						
18 ~ 29 years	121 (24.2)	61 (43.6)	17 (13.9)	40 (37.4)	21 (23.3)	1 (1.0)
30 ~ 39 years	196 (39.2)	59 (42.1)	39 (32.0)	37 (34.6)	42 (46.7)	50 (49.5)
40 ~ 49 years	157 (31.4)	15 (10.7)	62 (50.8)	18 (16.8)	19 (21.1)	50 (49.5)
≥ 50 years	26 (5.2)	5 (3.6)	4 (3.3)	12 (11.2)	8 (8.9)	0 (0)
Education level						
Secondary Education	44 (8.8)	20 (14.3)	6 (4.9)	5 (4.7)	1 (1.1)	4 (4.0)
Undergraduate education	415 (83.0)	104 (74.3)	107 (87.7)	87 (81.3)	74 (82.2)	97 (96.0)
Postgraduate education	41 (8.2)	16 (11.4)	9 (7.4)	15 (14.0)	15 (16.7)	0 (0)

*CHB* chronic hepatitis B, *GAD* Generalized anxiety disorder

Table 2 shows Spearman’s correlation coefficients of the total sample between the EQ-5D-5L domains and the SWEMWBS domains. Dimensions MO, SC, UA and PD of the EQ-5D-5L showed weak correlations with all dimensions of the SWEMWBS with the correlation coefficients ranging from 0.001 to -0.294 except that dimensions PD of the EQ-5D-5L showed moderate (*ρ* = -0.344) correlations with dimensions OP of the SWEMWBS. As expected, the dimension AD of the EQ-5D-5L showed a moderate correlation with dimensions OP (*ρ* = -0.496), USE (*ρ* = -0.396), RE (*ρ* = -0.483), PR (*ρ* = -0.361), CL (*ρ* = -0.331), CLO (*ρ* = -0.400), and MI (*ρ* = 0.353) of the SWEMWBS, respectively.

**Table 2:** Correlation (Spearman) between baseline domain scores for SWEMWBS and EQ-5D-5L

EQ-5D-5L	SWEMWBS						
	OP	USE	RE	PR	CL	CLO	MI
MO	-0.185**	-0.156**	-0.111*	0.073	0.089*	-0.065	0.001
SC	-0.127**	-0.146**	-0.105*	0.053	0.077	-0.085	-0.033
UA	-0.234**	-0.189**	-0.190**	-0.039	-0.032	-0.150**	-0.085
PD	-0.344**	-0.294**	-0.287**	-0.225**	-0.216**	-0.252**	-0.226**
AD	-0.496**	-0.396**	-0.483**	-0.361**	-0.331**	-0.400**	-0.353**

Correlation: 0.10–0.29 = small, 0.30–0.49 = medium, > 0.50 = large

*MO* Mobility, *SC* Self-care, *UA* Usual activities, *PD* Pain/discomfort, *AD* Anxiety/depression, *OP* Feeling optimistic about the future, *USE* Feeling useful, *RE* Feeling relaxed, *PR* Dealing with problems well, *CL* Thinking clearly, *CLO* Feeling close to other people, *MI* Able to make up my own mind about things

^*^Correlation is significant at the 0.05 level (2-tailed)

^**^Correlation is significant at the 0.01 level (2-tailed)

**Fig. 1:**
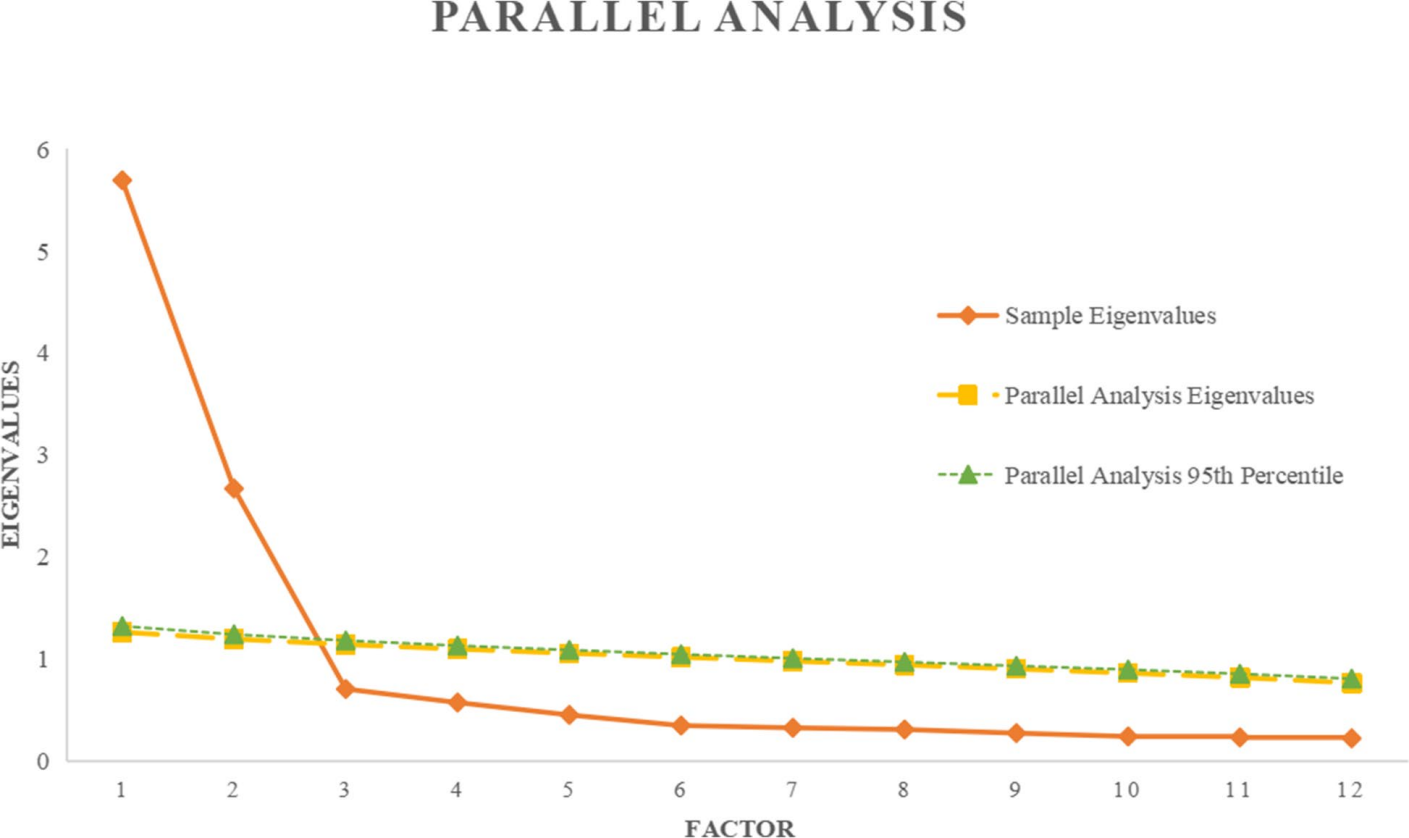
The parallel analysis of all items from both the SWEMWBS (7 items) and EQ-5D-5L (5 items)

In this study, the KMO was 0.910, indicating that the sample was adequate for performing factor analysis, and the Bartlett’s sphericity test was approximately χ2 = 4029.67, DF = 66, *P* < 0.001, indicating that the relationship among the variables was strong and the data were suitable to run an EFA [50]. The results of the EFA were shown in Table 3. Considering a scree plot, parallel analysis (Fig. 1) and the number of eigenvalues bigger than one, a two-factor solution was observed to be optimal which indicated that two separate, but correlated factors are evaluated by the pooled items of EQ-5D-5L and the SWEMWBS. The majority of EQ-5D-5L items (MO, SC, UA and PD) loaded onto factor 2 with a factor loading from 0.741 of PD item to 0.896 of MO item, while all of SWEMWBS items loaded onto factor 1 with a factor loading from 0.816 of OP item to 0.863 of PR item. These two factors explained 47.4% and 22.2% of variance respectively. The EQ-5D-5L item of AD loaded onto both factors (factor one = -0.608; factor 2 = 0.593).

**Table 3:** Exploratory factor analysis comparing the SWEMWBS and EQ-5D-5L items

	Factor 1	Factor 2
**EQ-5D-5L**		
MO		0.896
SC		0.862
UA		0.885
PD		0.741
AD	-0.608	0.593
**SWEMWBS**		
OP	0.816	
USE	0.817	
RE	0.818	
PR	0.863	
CL	0.828	
CLO	0.837	
MI	0.825	

*MO* Mobility, *SC* Self-care, *UA* Usual activities, *PD* Pain/discomfort, *AD* Anxiety/depression, *OP* Feeling optimistic about the future, *USE* Feeling useful, *RE* Feeling relaxed, *PR* Dealing with problems well, *CL* Thinking clearly, *CLO* Feeling close to other people, *MI* Able to make up my own mind about things

**Fig. 2:**
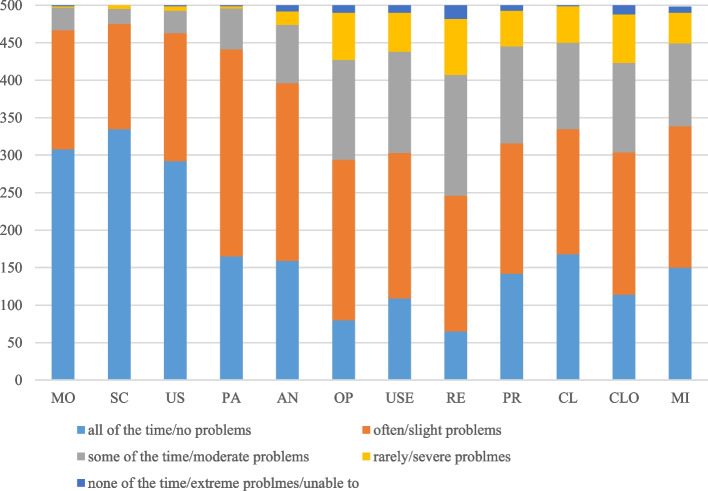
Response distributions of SWEMWBS and EQ-5D-5L. MO: Mobility; SC: Self-care; UA: Usual activities; PD: Pain/discomfort; AD: Anxiety/depression. OP: Feeling optimistic about the future; USE: Feeling useful; RE:Feeling relaxed; PR: Dealing with problems well; CL: Thinking clearly; CLO:Feeling close to other people; MI: Able to make up my own mind about things

Figure 2 shows the distributions of the EQ-5D-5L dimension and SWEMWBS dimension of the total sample. For the EQ-5D-5L, the majority of respondents reported ‘no problems’ in dimensions of MO (61.6%), SC (67.0%) and UA (58.4%). The EQ-5D-5L did not have many responses from level 4 and 5. The SWEMWBS had responses for all levels and the highest percentage was reporting ‘often’ in each dimension, especially in dimensions of OP, USE, CLO and MI, with 42.8%, 38.8%, 38.0% and 37.8%, respectively.

Table 4 shows the mean, standard error (SE), median and interquartile range (IQR) of the EQ-5D-5L utility scores, EQ-VAS and SWEMWBS scores. The total sample covered nearly all possible score ranges, with a mean of 0.82 (range =  − 0.31 to 1.0), 77.4 (range = 3–100) and 25.9 (range = 7–35), respectively. The mean scores of healthy group were 0.95(SE: 0.08), 83.1(SE: 0.23), 25.1(SE: 0.20), followed by HIV/AIDS of 0.78 (SE: 0.23), 84.7(SE: 0.13), 25.7(SE:0.17), CHB of 0.78(SE: 0.23), 73.7(SE: 0.25), 23.7 (SE: 0.18), depression of 0.75 (SE: 0.27), 67.1 (SE: 0.34), 21.3(SE: 0.25) and GAD of 0.72 (SE: 0.30), 63.9 (SE: 0.37), 20.7(SE: 0.26). In general, the median scores of healthy group were higher than four disease groups. Table 4 also reveals the median responses to EQ-5D-5L and SWEMWBS dimensions. For the EQ-5D-5L, the median response of the healthy group was ‘no problems’ across the five dimensions and ‘slight problems’ for HIV/AIDS. The median responses of CHB, depression and GAD were ‘no problems ‘across the two dimensions of ‘mobility’ and ‘self-care’, with mostly ‘slight problems’ across other three dimensions. For the SWEMWBS, the median responses of the healthy group were ‘often’ for all dimensions, while the median responses for depression and GAD were ‘some of the time’. The median responses of CHB and HIV/AIDS were mostly ‘often’ across all dimensions. The Mann–Whitney results were mostly significant at 0.01 level suggesting patients of four disease groups had different distributions of responses against the healthy group for both EQ-5D-5L and SWEMWBS.

**Table 4:** Score distribution of the EQ-5D-5L and SWEMWBS

		**EQ-5D-5L**							**SWEMWBS**							
		MO	SC	UA	PD	AD	Utility score	EQ-VAS	OP	USE	RE	PR	CL	CLO	MI	Score
Total (*N* = 500)	Mean(SE)	-	-	-	-	-	0.82(0.22)	77.4(0.25)	-	-	-	-	-	-	-	23.9(0.21)
	Median(IQR)	1	1	1	2	2	0.85(0.73–0.95)	82.2(70.0–90.3)	4	4	3	4	4	4	4	24.1(20.0–28.1)
Healthy (*N* = 140)	Mean(SE)	-	-	-	-	-	0.95(0.08)	83.1(0.23)	-	-	-	-	-	-	-	25.1(0.20)
	Median(IQR)	1	1	1	1	1	1(0.90–1.00)	89.5(80.0–95.3)	4	4	4	4	4	4	4	25.0(21.5–28.1)
CHB (*N* = 122)	Mean(SE)	-	-	-	-	-	0.78(0.23)	73.7(0.25)	-	-	-	-	-	-	-	23.7(0.18)
	Median(IQR)	1	1	2	2	2	0.82(0.73–0.89)	79.2(69.2–85.8)	4	4	3	4	4	4	4	23.2(20.3–27.0)
	*P*	< 0.01										0.484	0.787	0.337	0.166	0.034
Depression (*N* = 107)	Mean(SE)	-	-	-	-	-	0.75(0.27)	67.1(0.34)	-	-	-	-	-	-	-	21.3(0.25)
	Median(IQR)	1	1	2	2	2	0.78(0.70–0.89)	72.3(54.6–85.0)	3	3	3	3	3	3	3	20.0(17.4–24.3)
	*P*	< 0.01														
GAD (*N* = 90)	Mean(SE)	-	-	-	-	-	0.72(0.30)	63.9(0.37)	-	-	-	-	-	-	-	20.7(0.26)
	Median(IQR)	1	1	2	2	3	0.76(0.66–0.89)	67.9(49.5–84.7)	3	3	3	3	3	3	3	19.3(16.9–24.1)
	*P*	< 0.01														
HIV/AIDS (*N* = 101)	Mean(SE)	-	-	-	-	-	0.78(0.15)	84.7(0.13)	-	-	-	-	-	-	-	25.7(0.17)
	Median(IQR)	2	2	2	2	2	0.78(0.73–0.84)	89.9(80.3–91.6)	4	4	3	5	5	4	5	27.0(23.7–29.3)
	*P*	< 0.01						0.514	< 0.01	< 0.01	0.06	< 0.01	< 0.01	0.318	< 0.01	0.051

*SE* standard error, *IQR* interquartile range, *CHB* chronic hepatitis B, *GAD* Generalized anxiety disorder, *MO* Mobility, SC Self-care, *UA* Usual activities, *PD* Pain/discomfort, *AD* Anxiety/depression, *OP* Feeling optimistic about the future, *USE* Feeling useful, *RE* Feeling relaxed, *PR* Dealing with problems well, *CL* Thinking clearly, *CLO* Feeling close to other people, *MI* Able to make up my own mind about things

The results of the efficiency of the EQ-5D-5L and SWEMWBS are shown in Table 5. The F statistic of the EQ-5D-5L utility scores ranged from 92.19 of the CHB group to 179.05 of HIV/AIDS group, EQ-VAS ranged from 0.62 of HIV/AIDS group to 46.44 of the GAD group, and SWEMWBS scores ranged from 0.99 of HIV/AIDS group to 39.50 of the GAD group. The AUROC value of the EQ-5D-5L utility scores ranged from 0.81 of the CHB group to 0.92 of HIV/AIDS group, EQ-VAS ranged from 0.53 of HIV/AIDS group to 0.77 of the GAD group, and SWEMWBS scores ranged from 0.43 of HIV/AIDS group to 0.74 of the GAD group. It was clear that both EQ-5D-5L and SWEMWBS demonstrated good known-group validity, except that the EQ-VAS and SWEMWBS did not show a statistically significant result in the comparison of the healthy and HIV/AIDS groups. The Pearson correlation coefficient between the EQ-5D-5L utility scores, SWEMWBS scores and EQ-VAS was 0.44 (*P* < 0.01) and 0.65 (*P* < 0.01), respectively, indicating a moderate correlation.

**Table 5:** Efficiency of the EQ-5D-5L utility, EQ-VAS and SWEMWBS score

Comparison		Healthy	vs CHB	vs Depression	vs GAD	vs HIV/AIDS
	n	140	122	107	90	101
EQ-5D-5L Utility scores	F		92.19	115.62	129.03	179.05
	P		< 0.001	< 0.001	< 0.001	< 0.001
	AUROC		0.81	0.88	0.90	0.92
	95% Cl		(0.76, 0.87)	(0.84, 0.92)	(0.86, 0.94)	(0.88, 0.96)
EQ-VAS	F		16.73	36.97	46.44	0.62
	P		< 0.001	< 0.001	< 0.001	0.433
	AUROC		0.72	0.75	0.77	0.53
	95% Cl		(0.65, 0.78)	(0.69, 0.81)	(0.71, 0.83)	(0.45, 0.60)
SWEMWBS scores	F		5.69	33.07	39.50	0.99
	P		0.018	< 0.001	< 0.001	0.32
	AUROC		0.58	0.71	0.74	0.43
	95% Cl		(0.51, 0.65)	(0.64, 0.77)	(0.67, 0.81)	(0.35, 0.50)

*CHB* chronic hepatitis B, *GAD* Generalized anxiety disorder, *MO* Mobility, *SC* Self-care, *UA* Usual activities, *PD* Pain/discomfort, *AD* Anxiety/depression, *OP* Feeling optimistic about the future, *USE* Feeling useful, *RE* Feeling relaxed, *PR* Dealing with problems well, *CL* Thinking clearly, *CLO* Feeling close to other people, *MI* Able to make up my own mind about things

## Discussion

This study examined the relationship between SWEMWBS and EQ-5D-5L and compared their psychometric properties in 4 chronic conditions including CHB, depression, GAD and HIV/AIDS in China. The items of SWEMWBS did not show strong correlations with EQ-5D-5L items, indicating these two instruments measured different constructs. The two-factor solution for EFA supported the conclusion as their items loaded on different factors with the exception that AD dimension of EQ-5D-5L also loaded on the 1^st^ factor with all other SWEMWBS items. In terms of measurement properties, all five responses levels were used in EQ-5D-5L and SWEMWBS. No ceiling effects and floor effects were found in both instruments. Both EQ-5D-5L and SWEMWBS showed good known-group validity and convergent validity, with EQ-5D-5L having stronger discriminative ability but SWEMWBS having a higher correlation with EQ-VAS. Our study showed that both EQ-5D-5L and SWEMWBS were valid instruments measuring different constructs.

Dimensions MO, SC, UA, and PD of the EQ-5D-5L showed weak correlations with all dimensions of the SWEMWBS, suggesting good discriminant validity of both instruments. This was confirmed by the EFA results that these two instruments mainly loaded on different factors. The two-factor solution for the EFA found that all SWEMWBS items loaded onto the first factor, which could be interpreted as ‘mental well-being’, and all five EQ-5D-5L items loaded onto the second factor, which could be characterized as ‘physical health’. Notably, AD dimension from EQ-5D-5L loaded onto physical health factor was consistent with the research in Chinese type 2 diabetes patients conducted by Yao Xiong et al. [51], where the anxiety/depression dimension was loaded onto the same factor with the four dimensions of the EQ-5D. Xun Ran et al. also found that all dimensions except for the self-care dimension of EQ-5D loaded onto the same factor with the physical health and mental health dimensions of Well-being of Older People (WOOP) [52], which indicated anxiety/depression dimension including physical health and mental health. Our study indicated that the EQ-5D-5L and SWEMWBS are measuring two different constructs and therefore provided largely unique and complementary information, that is, the SWEMWBS measures positive mental well-being as intended, while the EQ-5D-5L measures HRQoL which covers both mental well-being and physical health. Since anxiety/depression also loaded on the mental well-being factor, it indicated that EQ-5D is able to measure both mental well-being and physical health.

Both the EQ-5D-5L and SWEMWBS showed good known-group validity and acceptable convergent validity results. As expected, the mean utility score of EQ-5D-5L and EQ-VAS for the healthy group were higher than the scores of the four disease groups, indicating good known-group validity. The F statistic and AUROC value of the EQ-5D-5L utility scores were significantly higher than the SWEMWBS scores in all four comparisons, which demonstrated that the EQ-5D-5L has a stronger discriminative ability in these four conditions groups that covered two chronic physical conditions and two mental conditions. The validity of EQ-5D-5L had been widely proven in previous studies [53–56]. The poorer efficiency of SWEMWBS differentiating healthy group and four disease groups was expected because SWEMWBS only measures positive mental well-being and there lacked research examining how health conditions could affect one’s positive mental well-being. The validity of SWEMWBS had also been widely reported in China, for example, Sun et al., Ng et al. and Fung reported that the Chinese SWEMWBS showed good validity and reliability for measuring mental well-being in the general population and populations with mental conditions [10, 12, 13]. However, more work is still needed to assess the sensitivity and test–retest reliability of the SWEMWBS measure.

The Pearson correlation coefficients between the EQ-5D-5L utility scores, SWEMWBS scores, and EQ-VAS showed a moderate correlation, indicating satisfactory convergent validity for both instruments. Remarkably, SWEMWBS had a larger correlation with EQ-VAS score, which measures a broader underlying construct of health [57]. Since SWEMWBS measures only positive mental well-being and EQ-5D-5L measures HRQoL that covers both physical and mental health, we would expect EQ-5D-5L to have a higher correlation with EQ-VAS, but this was not the case from our results. A possible explanation is that the utility of EQ-5D-5L represents the preference of the general public and both EQ-VAS and the SWEMWBS score represent the views of the respondent [58].

There are some limitations for this study. First, the study sample was young and highly educated. It should be due to the fact that old people and less educated people are less active on the Internet. Therefore, the findings of this study may not be generalizable to older populations. Second, some respondents reported more than one condition, but we did not provide a deep analysis about the possible effect of multi-conditions. It should be noted that our sample was recruited online and the health condition was self-reported. Ideally, clinical data is used to verify the presence and absence of diagnoses reported by the study subjects. Besides, the online survey might not bring good validity because we were not sure whether the samples completed the questionnaire by themselves. Last but not least, this study focused on analyzing how these two measures were associated and their psychometric properties. There may be a more complex relationship between positive mental well-being and PROM, as it may be hypothesized that respondents with positive mental well-being are more likely to cope with health problems and report no problems for PROM measures like EQ-5D-5L. Future studies should investigate this.

## Conclusions

SWEMWBS and EQ-5D-5L measure different constructs and can be seen as complementary measures. Both measures demonstrated good convergent validity and known-group validity with EQ-5D-5L being a more sensitive measure, even for mental conditions.

## Data Availability

Not applicable.

## Abbreviations

HRQoL: Health-related quality of life
WHO: World Health Organization
MO: Mobility
SC: Self-care
UA: Usual activities
PD: Pain/discomfort
AD: Anxiety/depression
PROM: Patient reported outcome measure
WEMWBS: Warwick-Edinburgh Well-Being Scale
SWEMWBS: Short Warwick-Edinburgh Mental Well-being Scale
CHB: Chronic hepatitis B
GAD: Generalized anxiety disorder
E-QALY: Extending the quality-adjusted life year
EQ-VAS: EQ visual analogue scale
OP: ‘I’ve been feeling optimistic about the future
USE: ‘I’ve been feeling useful
RE: ‘I’ve been feeling relaxed
PR: ‘I’ve been dealing with problems well
CL: ‘I’ve been thinking clearly
CLO: ‘I’ve been feeling close to other people
MI: ‘I’ve been able to make up my own mind about things
LSS: Level summary score
EFA: Exploratory factor analysis
KMO: Kaiser–Meyer–Olkin
SE: Standard error
IQR: Interquartile range
AUROC: Area under the receiver-operating characteristics curve
SE: Standard error; IQR: interquartile range
